# Regeneration performance of clay-based adsorbents for the removal of industrial dyes: a review

**DOI:** 10.1039/c8ra04290j

**Published:** 2018-07-10

**Authors:** Momina, Mohammad Shahadat, Suzylawati Isamil

**Affiliations:** School of Chemical Engineering, Universiti Sains Malaysia, Engineering Campus Nibong Tebal Pulau Pinang 14300 Malaysia chsuzy@usm.my mdshahadat93@gmail.com; Department of Biochemical Engineering and Biotechnology, Indian Institute of Technology, IIT Delhi Hauz Khas New Delhi-110016 India; Department of Textile Technology, Indian Institute of Technology, IIT Delhi Hauz Khas New Delhi-110016 India

## Abstract

The present review covers the regeneration capacity and adsorption efficiency of different adsorbents for the treatment of industrial dyes to control water pollution. Various techniques and materials have been employed to remove organic pollutants from water; however, adsorption techniques using cost-effective, ecofriendly, clay-supported adsorbents are widely used owing to their simplicity and good efficiency. Among all the natural adsorbents, activated carbon has been found to be the most effective for dye adsorption; however, its use is restricted due to its high regeneration cost. Clays and modified clay-based adsorbents are the most efficient clarifying agents for organic pollutants as compared to activated carbon, organic/inorganic, and composite materials. Regeneration is an important aspect to stimulate the adsorption efficiency of the exhausted/spent adsorbent for water treatment. A number of techniques, including chemical treatment, supercritical extraction, thermal, and photocatalytic and biological degradation, have been developed to regenerate spent or dye-adsorbed clays. This review discusses how these techniques enhance the adsorption and retention potential of spent low-cost adsorbents and reflects on the future perspectives for their use in wastewater treatment.

## Abstract 2

The present review covers the regeneration capacity and adsorption efficiency of different adsorbents for the treatment of industrial dyes to control water pollution.

## Graphical abstract

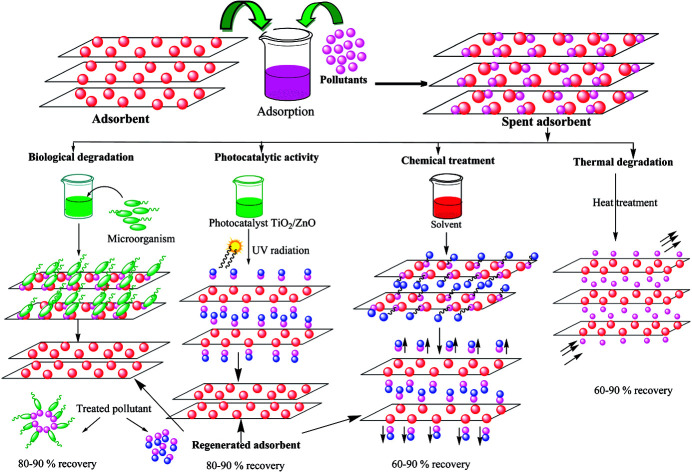

## 1. Introduction

Water is recognized as a vital material for all known forms of life, from early origins of life to advanced human civilization.^1^ It continuously shifts through various cycles, involving transpiration, condensation, evaporation, precipitation, and overflow, to reach water bodies. A huge content of water is combined with hydrated minerals on earth and is essential to living beings.^2^ In most parts of the world, much attention has been paid to accessing safe drinking water over the past decades; however, approximately one billion people are still short of access to safe drinking water and more than 2.5 billion people require water for sufficient sanitation.^3^ Based on the present scenario, it is expected that the world population may rise to 9 billion by 2050, which will put even greater demands on access to water (with a shortage of fresh water).^4^ So the treatment of water is mandatory for sustaining the life of living beings.^5^ Industrial effluents and agricultural pesticides are some culprits that have become important sources and causes of water pollution.^6^ The discharge of these effluents, even in a small concentration, into water bodies poses a great threat to fresh water as well as to aquatic animals, resulting in severe disturbances to ecological systems.

The consumption of pollutants-containing water poses a risk of wastewater-borne diseases, which have a direct effect on the environment and human health.^7^ Dyes have been used as coloring agents in the textile industries for many years. More than 100 000 dyes are commercially available and approximately 7 × 10^5^ tons of dyes and their derivatives are produced annually.^8,9^ Because of their complex structure and typically synthetic origin, the decolorization of dyes is difficult.^10,11^ Dyes are non-biodegradable molecules having a carcinogenic action or causing allergies, dermatitis, or skin irritation due to their toxic nature.^12^

Different treatment techniques and materials, including adsorption,^13,14^ biological treatment,^15,16^ oxidation,^17^ ion exchange,^18–20^ organic resin,^21^ filtration,^22^ precipitation,^23^ electrolysis,^24^ reverse osmosis,^25^ and coagulation,^26^ biofoulents^27^ biodegradable nanocomposites,^28^ adsorbant coatings,^29^ and hybrid materials^28,30^ have been employed to remove dyes from wastewater. Since synthetic dyes cannot be efficiently decolorized by traditional methods (*e.g.*, activated sludge process, coagulation, oxidation). However, adsorption is strongly favored over the other techniques to remove dyes from wastewater because of its simplicity, cost effectiveness, ease of operation, and good efficiency.^31^ In addition, proper adsorption has the potential to produce a high-quality treated effluent.^32^

**Fig. 1:**
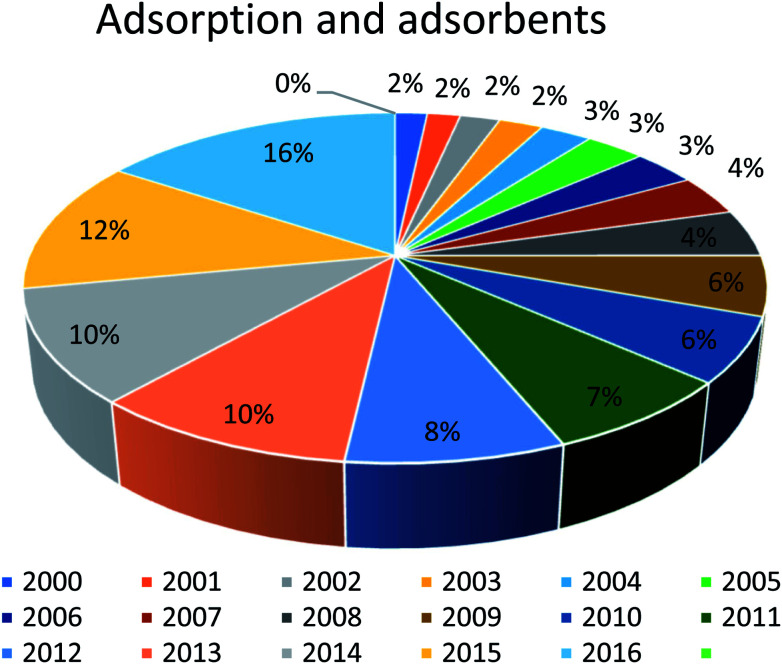
Published reports on the treatment of industrial dyes using different adsorbents as a percentage of the reports published on this topic per year since 2000.

In this regard, several adsorbents are often used; however, the choice of adsorbents depends on many factors (concentration and type of micropollutant, its efficiency/cost ratio, adsorption capacity, high selectivity for a large volume of water). Moreover, these adsorbents should be nontoxic, low cost, re-generable, easily recoverable from filters, readily available, and should lead to zero waste/sludge.^33^ The most common commercially available adsorbents are activated carbon, ion-exchange materials, biosorbents, zeolite, bentonite clay, *etc.* There are numerous studies in the literature related to the adsorption behavior of adsorbents for the removal of pollutants, as shown in Fig. 1. It can be inferred from the results of the reported data that work on adsorption continuously increased from 2000 to 2016 and still has a tremendous potential for improvement. Different developed methodologies are still improving the adsorption efficiency in terms of achieving a high sorption efficiency to remove pollutants from wastewater, and for regenerative of the adsorbent, and most importantly toward a more cost-effective wastewater treatment. The present review highlights the various types of adsorbents widely used for the removal of dyes from wastewater. Furthermore, different regeneration methods of clay adsorbents, including chemical, thermal, supercritical extraction, and photocatalytic and biological degradation and their future perspectives are discussed.

### 1.1. Adsorbent efficiency of different adsorbents

#### 1.1.1. Activated carbon

The removal of dyes from wastewater using activated carbon (AC) has been found to be effective and is more extensively studied as compared to the other adsorbents. Indeed, AC is probably the most versatile adsorbent owing to its large surface area (>600 m^2^ g^−1^), polymodal porous nature, and high adsorption capacity.^34^ Activated carbon-based adsorbents can be prepared from coal, coconut shells, peanuts, lignite, wood, *etc.* using physical or chemical activation methods. However, due to its typical high cost, the use of AC is restricted and the developed techniques (chemical and thermal) are quite expensive for its regeneration.^35^ Furthermore, these techniques affect the structure of the activated carbon, resulting in a slightly lower adsorption capacity as compared to virgin activated carbon.^36^ Therefore, researchers have sought to prepare low-cost adsorbents that could replace activated carbon to control pollution through an adsorption process.^37^ Some reported commercially activated carbon-based adsorbents used for the removal of dyes from wastewater are listed in Table 1a.

**Table 1:** Adsorption efficiencies of different adsorbents, (a) adsorption efficiency of activated carbon for dye pollutants, (b) adsorption performance of bioadsorbents, (c) adsorption capacities of agriculture and industry wastes, (d) adsorption capacities of zeolites, (e) adsorption capacities of clay-based adsorbents

Adsorbents	Targeting species	Adsorption capacity (mg g^−1^)	Reference
**(a) Adsorption efficiency of activated carbon for dye pollutants**			
Granular activated carbon	Acid yellow	1179.0	106
		133.3	107
Cocoa pod husk	Remazol black B	22.1	108
Activated carbon Filtrasorb 400	Remazol yellow	1111.0	109
Commercial activated carbon	Methylene blue	980.3	103
Peat		324.0	110
Wheat straw		312.5	111
*Posidonia oceanic* L.		285.7	112
Granular activated carbon		57.47	107
Rambutan peel	Malachite green	404.5	113

**(b) Adsorption performance of bioadsorbents**			
Chitosan (bead, lobster)	Reactive red 222	1037.0	40
Chitosan (flake, crab)		293.0	
*Rhizopus arrhizus* biomass	Reactive black 5	588.2	45
*Spirodela polyrrhiza* biomass	Basic blue 9	144.93	114
Activated sludge biomass		256.41	115
Crosslinked chitosan bead	Reactive red 2	1936.0	116
Yeasts	Remazol blue	173.1	117

**(c) Adsorption capacities of agriculture and industry wastes**			
Raw date pits	Methylene blue	80.29	59
Papaya seeds		555.55	61
Fly ash (bagasse)		6.46	66
Red mud		2.49	67
Orange peel	Methyl orange	20.5	62
Metal hydroxide sludge	Reactive red 2	62.5	65
	Reactive red 141	56.18	
Bark	Basic red 2	1119.0	118
Teak wood bark	Methylene blue	914.59	118
Rice husk	Basic red 2	838.0	
Cedar sawdust	Methylene blue	142.36	119
Meranti sawdust		120.48	120
Cherry sawdust		39.84	121
Red mud	Direct red 28	4.05	122

**(d) Adsorption capacities of zeolites**			
Zeolite	Basic dye	55.8	123
	Methylene blue	53.1	124
	Reactive yellow 176	11.8	125
	Methylene blue	10.8	126

**(e) Adsorption capacities of clay-based adsorbents**			
Moroccan natural clay	Malachite green	81.22	79
	Methylene blue	56.25	
Bentonite	Methylene blue	1667.0	127
Dodecyltrimethylammonium bromide-modified bentonite	Acid blue 193	740.5	128
Montmorillonite	Methylene blue	289.12	129
Bentonite	Basic red 2	274	105
	Methylene blue	151–175	130
Kaolinite	Malachite green	52.91	131
Modified montmorillonite	Methyl orange	24.0	132
Bentonite	Reactive black 5	13.07	133

#### 1.1.2. Bioadsorbents

Currently, natural adsorbents, such as chitin, chitosan, and biomass, and waste materials from industry and agriculture are used to treat dye effluents. Bioadsorbents are more selective, cheaper, and efficient than traditional ion-exchange resins and commercially activated carbon and can reduce the dye concentration down to the ppb level.^32^ Chitin and chitosan have a high affinity toward acidic dyes as compared to basic dyes.^38^ Various studies for the removal of cationic dyes using chitosan are listed in Table 1b. Chitosan-based adsorbents are so versatile that they can be used in the form of beads, flakes, and gels. Among all the forms, the beads form of chitosan exhibit excellent performance toward the adsorption of anionic dyes in comparison to activated carbon (the adsorption values of beads were found to be 3–15 times higher than activated carbon at the same pH).^39^

To get an idea regarding the sorption mechanism of chitosan adsorbents different kinds of interaction (*e.g.*, ion-exchange interactions, hydrophobic attraction, physical adsorption) have been studied.^39,40^ Wu *et al.* (2000) revealed that intraparticle diffusion plays an important role in the sorption mechanism.^40^ The major adsorption or active site of chitosan is due to the existense of a primary amine group, which provides a strong electrostatic interaction between the amine groups and dye molecules ensuring effective sorption.^39^

The effect of the stirring rate on the adsorption mechanism of Acid Blue 9 and Food Yellow 3 onto chitosan has been investigated.^41^ The results suggested that adsorption was a chemical process and occurs through internal and external mass transfer mechanisms. During adsorption, stirring also plays a key role. Increasing the stirring rate from 15 rpm to 400 rpm increased the adsorption capacity of chitosan powder for Acid Blue 9 and Food Yellow 3 by 50% and 60%, respectively. The stirring rate increased the film diffusivity, while the adsorption capacity increased with the increasing intraparticle diffusivity. However, some other factors, such as pH, contact time, or flux, also affect the sorption capacity. At low pH, free amino groups of chitosan are protonated, which can be easily attracted with dyes molecules, ensuring higher adsorption.^42^ Despite its good efficiency, some disadvantages are associated with chitosan. The adsorption properties of chitosan depends on the degree of *N*-acetylation, molecular weight, solution properties, and vary with crystallinity, affinity for water, and percent deacetylation as well as the amino group content.^32,43,44^

Recently, biosorption has become an emerging technology that attempts to overcome the selectivity disadvantage of conventional adsorption.^32^ It provides an alternative to existing technologies because it is more cost effective and ecofriendly without huge production of sludge. The use of biomass is increasing because of its low cost and availability on a large scale and due to its ecofriendly nature. Large numbers of by-products are generated from the fermentation process and have been used as bioadsorbents for the removal of pollutants. Algae, fungi, and other microbial cultures are used for decolorizing dyes with a high efficiency (Table 1b). The treatment of dyes using *Rhizopus arrhizus* has been found to be effective as compared to activated carbon and other alternative bioadsorbents.^45,46^

The use of biomass is especially interesting when the dye-containing effluent is very toxic. Moreover, bacterial decolorization is normally faster compared to fungal systems in the decolorization and mineralization of azo dyes.^47^ On the other hand, single individual bacterial strains are unable to degrade azo dyes completely, and intermediate carcinogenic products (aromatic and amines) are often obtained, which then need to be further decomposed.^48^ In microbial consortium, individual strains may attack the dye molecule at different positions or may utilize metabolites produced by the co-existing strains for further decomposition.^49,50^

The removal of dyes by bacterial decolorization depends on the sources of oxygen, carbon, and nitrogen, temperature, pH, dye concentration, and the electron donor and redox mediator.^47^ The decolorization of dyes by living or dead cells of biomass can be explained by several mechanisms (surface adsorption, ion exchange, complexation (coordination), complexation–chelation, and micro-precipitation). Dyes molecules interact with different groups (polysaccharides, proteins, and lipids) on the bacterial cell wall.^32^ Although, biomass has good adsorption characteristics and high selectivity; however, the sorption of biomass is quite slow and very few studies and limited practical applications of biomass have been examined. Biomass has been found to not be appropriate for the treatment of effluents using column systems due to their clogging effect.^32^

#### 1.1.3. Agriculture and industrial waste by-products and waste

##### 1.1.3.1 Agriculture waste

Raw agriculture solid waste (leaves, fibers, fruits peels, seeds, *etc.*) and waste materials from forest (sawdust and bark) are extensively used as adsorbents for the removal of dyes.^51^ These materials are available in enormous quantities and have sorbent potential due to their physicochemical characteristics. Some agricultural solid wastes can remove both types of dyes (cationic and anionic), although they need activation.^52,53^ The most important factor that affects dye-classified adsorption is the pH. A high pH is preferred to adsorb cationic dyes, while at low pH, anionic dyes are adsorbed.^54^

Sawdust has been used for the removal of dye pollutants from wastewater.^55^ The adsorption capacities of some reported sawdusts to treat industrial effluents are listed in Table 1c. The sorption mechanism is due to several interactions: complexation, ion exchange due to a surface ionization, and hydrogen bonds. Sawdust has been found to be strongly pH dependent—beyond neutral pH, it can act as an anion and cation.^56,57^ Therefore, the sorption capacity of a basic dye is much higher than that of acid dyes because of ionic charges on the dyes and the ionic character on sawdust. Bark is another waste polyphenol-rich product obtained from the timber industry. It is generally used as an adsorbent as a result of its low cost and high availability. On account of its high tannin content, bark has been found to be an effective adsorbent.^58^ Like sawdust, the cost of bark wastes is only associated with the transport cost from the storage place to the site of utilization (Table 1c). Other agricultural solid wastes, such as date pits,^59^ barley husk,^60^ papaya seeds,^61^ orange peel,^62^ neem,^63^ and corn-cob,^60^ have also been used as cost-effective, ecofriendly adsorbents to treat dye effluents.

##### 1.1.3.2 Industrial waste

Recently, the extension of industrialization has generated huge amount of solid waste in the form of by-products. Some of these are reused and the remaining disposed of in landfills. These industrial wastes are almost free of cost and cause a disposal problem;^64^ therefore, they can be reused as a cost-effective adsorbent. Metal sludge, fly ash, and red mud are some commonly used low-cost adsorbents obtained from industrial waste for the removal of dyes^65–67^ (as shown in Table 1c).

Metal hydroxide-based sludges are dried waste produced by the precipitation of metal ions in calcium hydroxide in electroplating industries. They are positively charged adsorbents and show high adsorption capacity for azo reactive (anionic) dyes.^65^ Fly ash is obtained from their combustion and contains toxic metal elements.^68^ However, bagasse fly ash, obtained from the sugar industry, is free from any toxic metals and is widely used for the adsorption of dyes.^66^ Kumar and coworkers studied the removal adsorption mechanism of methylene blue using flyash.^69^ Red mud is another abundant industrial by-product^67^ from the Bayers process used for extracting alumina from bauxite ore.^70^ Red mud, can be used for the removal of MB.^67^

#### 1.1.4. Natural adsorbents

##### 1.1.4.1 Zeolites

Zeolites are commonly used as adsorbents and catalysts for the treatment of organic and inorganic pollutants. The structure of zeolites consists of a negatively charged lattice, which has an exchangeable potential to exchange cations in solutions. Zeolites are microporous, aluminosilicates with a high ion-exchange capacity, high specific surface area, rigid porous structure, and are cost effective, which makes them attractive adsorbents. Zeolite adsorbents have been effectively used in wastewater treatment as mentioned in Table 1d. The low permeability of zeolites means they require an artificial support to use in column operations. The sorption mechanism of zeolite particles was found to be complex because of their porosity, inner and outer surface charges, and mineralogical heterogeneity, and due to other imperfections on their surface.^71,72^ In comparison to other clay adsorbents (*e.g.*, clinoptilolite), the removal efficiency of zeolites (for dyes) is not as good as that of clay material, like raw clay, and they were found not to be suitable for the removal of reactive dyes due to their extremely low sorption capacities.^73,74^ However, their easy availability and low cost may compensate for their associated drawbacks to some extent.^36^ Therefore, new methods are required to increase the sorption capacity of zeolites by modifying them with quaternary ammonium salts,^75^ surfactants,^9^ or chitosan.^76^

##### 1.1.4.2 Clays

Clays are hydrous aluminosilicate minerals made up of the colloidal fraction (<2 μ) of soils, sediments, rocks, and water^77^ and are composed of mixtures of fine-grained minerals and clay-sized crystals of other minerals (*e.g.*, quartz, carbonate, and metal oxides). The prominent ions found on the clay surface are Ca^2+^, Mg^2+^, H^+^, K^+^, NH_4_^+^, Na^+^, SO_4_^2−^, Cl^−^, PO_4_^3−^, and NO^3−^. These ions can be easily exchanged with other ions without affecting the structure of the clay mineral.^78^ Because of the requirement of low-cost adsorbents for wastewater treatment, natural clays are well known from the earliest days of civilization and have been found to be effective for removing pollutants from wastewater. The adsorption efficiency of clays generally depends on the net negative charge of the mineral.^79^ The main reason for the high adsorption capacity of clays is their high surface area (ranging up to 800 m^2^ g^−1^).

Among clay-based adsorbents, bentonite is the most commonly used clay in water purification. It consists of montmorillonite and has excellent rheological and adsorptive properties^80,81^. Bentonite is characterized by a three-layer structure with two tetrahedral silicates layers covered by one central octahedral sheet of aluminate layer in a 2 : 1 ratio and having negative charges on its lattice. As a result, it could be assumed that bentonite has great affinity toward cationic dyes due to the attraction of opposite charges on the surface of the lattice.^80^ Isomorphous substitution results in various types of smectite and causes a net permanent charge balanced by cations in such a manner that water may move between the sheets of the crystal lattice, giving it reversible cation-exchange properties.^82^ Other commonly known clays are sepiolite and palygorskite, which are fibrous in nature, having the chemical formulas Si_12_Mg_8_O_30_(OH)_4_(H_2_O)_4_·8H_2_O and Si_8_Mg_5_O_20_(OH)_2_(H_2_O)_4_·4H_2_O, respectively.^83^ Because of their hollow and porous structure, these clays have significant potential for the retention of micropollutants, including heavy metals and cationic dyes.^80^ A significant adsorption efficiency of clay-based adsorbents has been achieved compared with activated carbon.^84^ Anionic dyes, such as acid yellow 194, acid blue 349, and acid red 423 can be removed by bentonite and sepiolite with good adsorption capacities (98.6, 99.9, and 95.2 mg g^−1^, respectively) compared with activated carbon (49.2, 68.2, and 26.3 mg g^−1^ respectively). Among these adsorbents, sepiolite showed a higher capacity for acidic blue than activated carbon but a comparable capacity to bentonite (24.9, 92.7, and 29.1 mg g^−1^, respectively).

### 1.2. Clay-based adsorbents

Industrial wastewaters have been treated for the removal of organic and inorganic pollutants using different adsorbents (organic, inorganic, hybrid, natural clays) and the work on adsorption has continuously increased in recent decades, as shown in Fig. 1. The selection of the adsorbent is carried out on the basis of high adsorption capacity toward dye pollutants in a short time. Higher dye removal capacities have been achieved by organic/inorganic or hybrid materials; however, the high cost and production of a huge amount of sludge after adsorption are serious issues in water treatment. Based on adsorption study results, it was found that among various adsorbents, clays and modified clays are commonly applied in dye treatment because of their low-cost and ecofriendly nature. Besides their good adsorption potential, clay-based adsorbents have good regeneration capacity. A number of reviews have reported the adsorption behaviors of organic/inorganic, composite hybrid, and nature-inspired materials;^10,27,32,36,54,85–101^ however, no review has yet focused on the regeneration potential together with the adsorption capacity of clay-based adsorbents. To improve the regeneration and adsorption efficiency of clay-supported adsorbents, the present authors were determined to write a review on low-cost adsorbents. Due to the large number of adsorbent studies, we have restricted ourselves to studies on clays as they are the cheapest material and are naturally available in huge abundance. As compared to other adsorbents, clay-based modified adsorbent materials show exceptional regeneration and adsorption capacities and selectivity, along with being low cost and having a porous nature and high surface area.

### 1.3. Adsorption mechanisms of clay-based adsorbents

The adsorption mechanism for the removal of dyes involves a number of steps, including: diffusion of the dye through the boundary layer, followed by intraparticle diffusion and finally adsorption of the dye on the sorbent surface.^102,103^ The adsorption of acid blue 193 onto benzyltrimethylammonium (BTMA)-bentonite was followed by intraparticle diffusion.^104^ Similarly the adsorption of acid red 57 (AR57), acid blue 294 (AB294), and congo red on acid-activated bentonite was found to occur by intraparticle diffusion. An outline for the adsorption of cationic dyes on the surface of clay-supported adsorbents is shown using the following equations:1
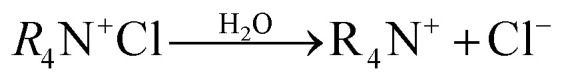
2
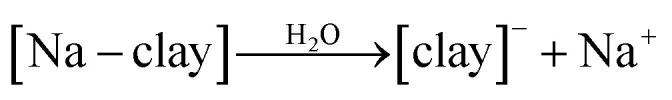

3 R_4_N^+^ + [clay]^−^ ↔ R_4_N − clay

In aqueous solution, the dye molecule (R_4_N^+^Cl) dissociates into its ions (ammonium cation and chloride anion as shown in eqn (1)). Addition of clay to the dye-containing aqueous releases exchangeable cations (sodium, calcium, hydrogen ions), leaving the clay surface with a negative charge (eqn (2)). At the same time, cationic dye molecules (basic red 2) are attracted by the negatively charged surface of the clay molecules (eqn (3)).^105^ Regarding the adsorption mechanism, no clear distraction is made to the uptake of dye molecules on the clay's surface, which may be partly attributed to ion-exchange complexion, a pore filling mechanism, or simply adsorption.

**Fig. 2:**
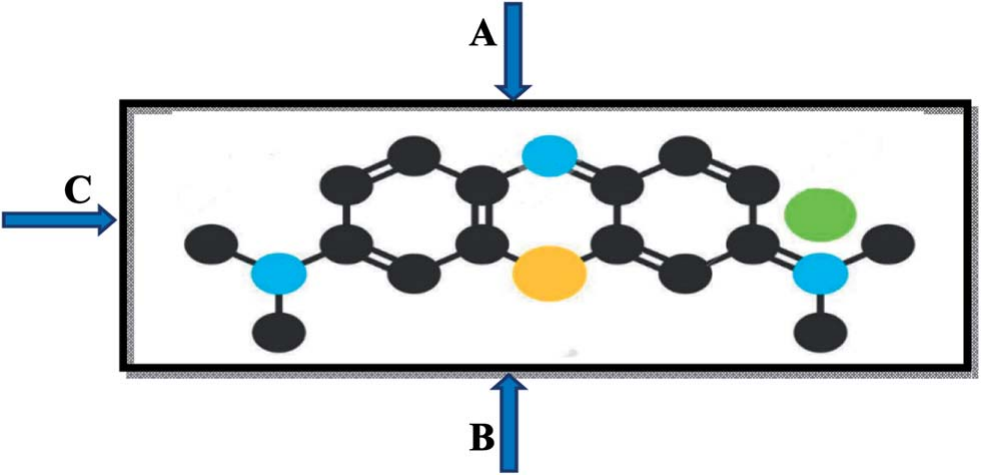
Possible orientation of dye molecule: (A) flat with maximum surfaces, (B) aligned along the longer axis, (C) aligned along the shorter axis.

Additionally, the pH of the dye solution also plays a main role during adsorption. Most of the dye adsorption is observed in the pH range 4–10. The pH affects the speciation of dye and clays, and makes adsorption feasible. The electrostatic force of attraction between charged dye molecules coupled with the concentration gradient on the adsorbent surface are the main driving forces to drive dye molecules on to the surface of the adsorbents. The aggregation of dye molecules flat on the clay surface is fast and represents the easiest mode of adsorption. At low dye concentration, slow adsorption is found, however, on increasing the concentration of dye, as gradient forces adsorbed molecules orient differently to accommodate more dye molecules on the surface under the influence of physical forces. Thus, adsorption increases so much so that it attains equilibrium (saturation). The three possible orientations for dye molecules to adsorb on the surface of an adsorbent are shown in Fig. 2(A–C).

Orientation A (flat with maximum surface) leads to minimum adsorption, followed by orientation B (aligned along the longer axis of the dye, leading to medium adsorption) and C (aligned along the shorter axis of the dye, leading to maximum adsorption). The orientation depends on the pH, the concentration of the dye, and the extent of the attractive forces between the dye and clay. The effects of these forces will not be an issue beyond one flat lying molecule *via* orientation A. In particular, interactions between dyes and clays have been extensively studied with better ion-exchange efficiency, as listed in Table 1e.

The adsorption of clays can be improved by modifying with acid,^134^ thermal treatment,^135^ polymer addition,^136^*etc.* Bentonite coating is an efficient methodology not only for durability and low cost, but also due to its wide acceptability in many industries. Bentonite-based adsorbents have been prepared by mixing bentonite, a water-based binder, and a solvent in a specific ratio to remove methylene blue from synthetic dye solution, achieving a higher adsorption capacity (99%).^137^ Adsorbent coatings have been used to overcome the problem associated with the use of adsorbents in pellet, beads, powder, or other particle forms, where they improve the catalytic and adsorption capacity of adsorbents by increasing the surface area/weight ratio. Additionally, such coating also reduces the quantity of solid adsorbent required, enhances the binding strength, protects the substrate from harmful environment, and performs a specific desorptive or catalytic role over the entire surface of the substrate.^138^

**Fig. 3:**
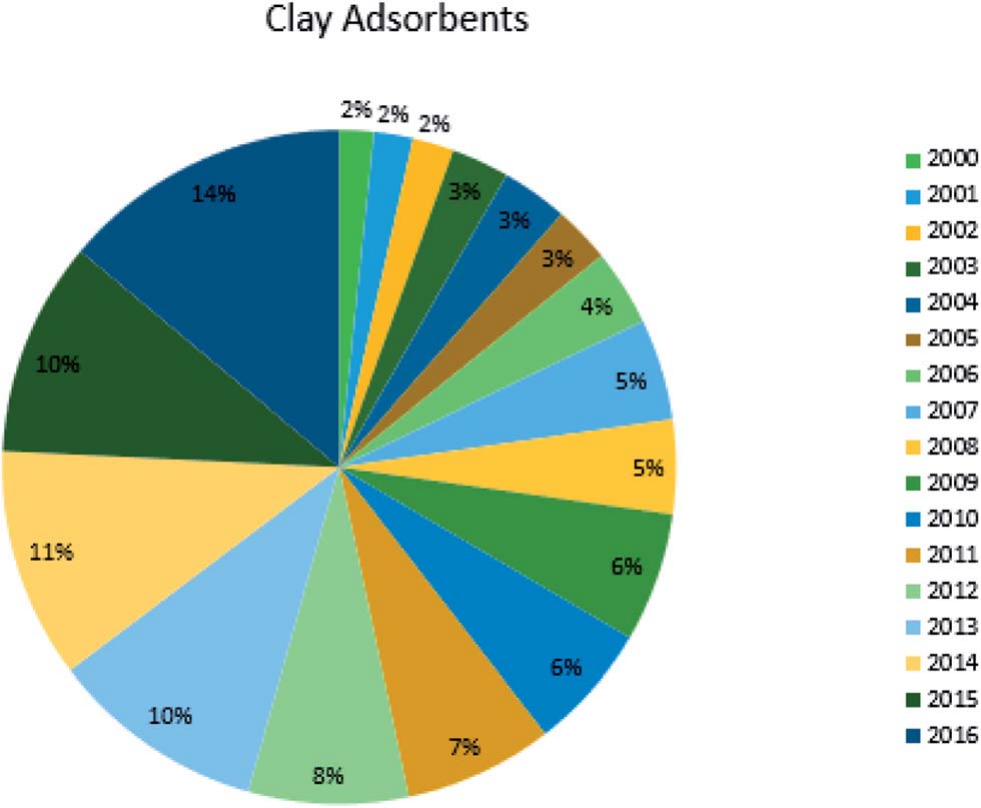
Published reports on the removal of dyes using clay-based adsorbents as a percentage of reports published on this topic per year since 2000.

Work on the adsorption of dyes using bentonite as an adsorbent has been increases exponentially over the past two decades, as shown in Fig. 3. The work carried out on clay adsorption from 2000 to 2016 shows the highest number of publications in 2016. The main reason for this improvement is the low cost, convenience, ease of operation, simplicity of design, and ecofriendly nature of clay adsorbents, which has consequently seen research interest in them increase. Rather than the expensive commercial activated carbon, clay minerals have been used for the effective removal of dyes from aqueous solution. However, a great deal of work still needs to be done to predict the performance of the clay to adsorb dyes in real-world industrial effluents under various operating conditions.

### 1.4. Cost comparison of adsorbents

**Fig. 4:**
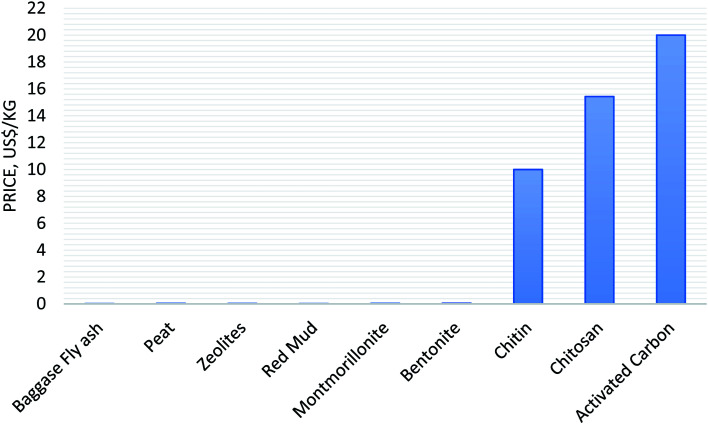
Cost of adsorbents as reported in the literature.^139,139–141^

The dye adsorption capacity of different adsorbents for the removal of dyes has been discussed in detail; however, selection of a low-cost adsorbent is another important factor to treat wastewater.^36^ The cost of the adsorbent depends on many factors, such as its availability, and source (natural, industrial/agricultural/domestic waste, by-products, or synthesized products), treatment conditions, recycle, stability, country of production (such as developed, developing, or under developed).^36,64^ Thus, a comparative study regarding the cost of adsorbents was carried out, as shown in Fig. 4. The comparative study data revealed that natural adsorbents (baggase fly ash, peat, zeolites, clay: montmorillonite and bentonite) have a low price < 1.0 US$ per kg, which makes them more useful adsorbents compared to high-cost activated carbon. However, the cost of other adsorbents (organic/inorganic composite, CNT-based hybrid adsorbents) was found to be approximately fourfold higher in price as compared to the cost of natural adsorbents. Thus, low-cost natural adsorbents have applicability in the treatment of industrial wastewaters.

## 2. Regeneration potential of clay-supported adsorbents

Regeneration is defined as the rapid recycling or recovery of spent adsorbents using technically and economically feasible methods. Since, cost is a crucial parameter for the development of new adsorbents, the regeneration of clays has immense importance for organic pollution control. A number of regeneration methods, including thermal regeneration, steam regeneration, pressure swing regeneration, vacuum regeneration, micro wave regeneration, ultrasound regeneration,^142^ chemical regeneration, oxidative regeneration, ozone regeneration, and bioregeneration,^143^ have been employed to retain the adsorption capacity of adsorbents. In some cases, the combined effects of these regeneration techniques (thermochemical regeneration, electrochemical, *etc.*) have been observed.^144^ For clay adsorbents, some regeneration methods are described below.

### 2.1. Chemical treatment

Chemical regeneration involves the desorption of a particular species using specific solvents and/or chemical species in solutions or by the decomposition of adsorbed species using chemicals that act as oxidants under supercritical or subcritical conditions.^145^ The regeneration capacity of any adsorbent depends on the solution pH, and the rate of oxidation and degradation by complexion.

#### 2.1.1. The effect of solution pH

The regeneration efficiency of an adsorbate or adsorbents can be retained by changing the solution pH in which adsorbed pollutant may exchange with a cation or anion. Commonly used reagents (NaOH, HCl, and acetone) have been employed for the regeneration of adsorbents by altering the solution pH and consequently the retention of the charged state of adsorbents or adsorbates. Sodium hydroxide has been used to desorb tannin^146^ and phenol^147^ from organoclays (Table 2). Anirudhan & Ramachandran (2006) established a 99% adsorption efficiency of tannin for organobentonite at pH 4, which remained almost the same after 2 regeneration cycles, however, the desorption efficiency was found to be decreased (from 99.7% to 89.3%) after four regeneration cycles using aqueous NaOH solution.^146^ Similarly, Yang and coworkers found decline in desorption efficiency of hexadecyltrimethylammonium (HDTMA)-modified montmorillonite (HMM) for the desorption of phenol using NaOH (as desorbing reagent).^147^ The desorption of methylene blue from clay-papaya seed composite adsorbents using aqueous HNO_3_ solution (0.001 to 0.1 M) showed 90% desorption efficiency throughout five consecutive regeneration cycles.^148^

**Table 2:** Removal efficiency of dyes from clay adsorbents using chemical desorption

Adsorbent	Adsorbate	Removal efficiency (%)	Solvent	Reference
Modified hydrotalcite	Safranine	85.0	Acetone	149
Organobentonite	Tannin	From 99.7–89.3	NaOH	146
HDTMA-modified montmorillonite	Phenol	—	NaOH	147
Clay-papaya seed	Methylene blue	90.0	HNO_3_	148

The effect of temperature was also examined and revealed that on increasing the temperature from 30 °C to 50 °C, the efficiency was reduced to 50%. Acetone can act as a strong solvate for organic compounds^149^ and has been used to regenerate modified hydrotalcite for the adsorption of basic dye (safranin) from aqueous solution. After two regeneration cycles, the removal efficiency of the dye was found to be the same as that of the original clay (85%). These solvents may alter the nature of the adsorbents by interacting with the constituents and damaging the structure, which results in a loss in adsorption capacity.^150^

#### 2.1.2. Fenton regeneration

In the Fenton regeneration, OH radicals are produced by the reaction of ferrous ions and H_2_O_2_, as shown in eqn (1).

4 Fe^2+^ + H_2_O_2_ → Fe^3+^ + ^−^OH + ˙OH

**Fig. 5:**
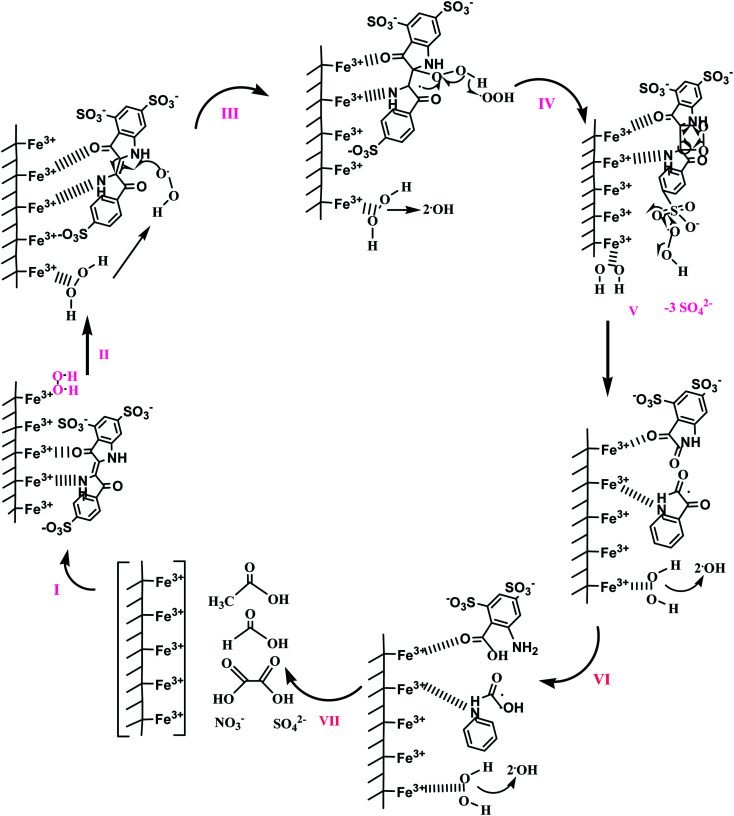
Proposed outline to stimulate regenerated iron- and copper-modified clay from indigo blue by using the photo-Fenton process. This figure has been adapted/reproduced from ref. 152 with permission from Elsevier.

The reaction pathway of this process includes the photoreduction of Fe^3+^ to Fe^2+^ and the subsequent re-oxidation of Fe^2+^ to Fe^3+^ by H_2_O_2_. The produced free radicals undergo secondary reaction and rapidly degrade organic compounds by releasing the super hydroxyl's power.^151^ Almazán-Sánchez *et al.* (2016) regenerated iron- and copper-modified clay from indigo blue by using a photo-Fenton process. A proposed mechanism of potassium indigo trisulfonate oxidation is shown in Fig. 5. Initially, chemisorption occurs for adsorption of the dye (Step I), followed by the formation of a hydroperoxyl radical (Step II). Further, it reacts with dye molecule leading to bond cleavage (Step III), followed by the formation of sulfate ions (Step IV) and 1*H*-indoline-2,3 dione (Step V); then it is oxidized to 2-(2-aminophenyl)-2-oxoacetic acid (Step VI). Finally, the probable oxidation products (oxalic, formic, acetic acid, sulfate, and nitrate ions) are formed. The removal efficiency of iron- and copper-modified clays was found to be 90% and this could maintained during four successive cycles.^152^ However, there were some drawbacks associated with the generation of oxidants wastage because of the self-decomposition of hydrogen peroxide, the continuous loss of iron ions, and the formation of a solid sludge. Flotron *et al.* (2005) reported several economic and environmental effects of Fenton oxidation.^153^

The solution pH also affects the efficiency of the photo-Fenton process. The existence of Fe^2+^ can foul the surface of photocatalysts through the formation of Fe(OH)_3_, while PO_4_^3−^ in a nominal pH range fouls the active sites of the TiO_2_ surface and inhibits its photoactivity.^154–156^ Therefore, photocatalytic regeneration needs to be improved in a wide range of solution pH to minimize the addition of oxygen additives, which produce secondary pollutants. Furthermore, the integration of different advanced oxidation processes (AOPs), such as the photo-Fenton, could be a feasible alternative to reduce costs without decreasing the efficiency.

### 2.2. Supercritical extraction

**Fig. 6:**
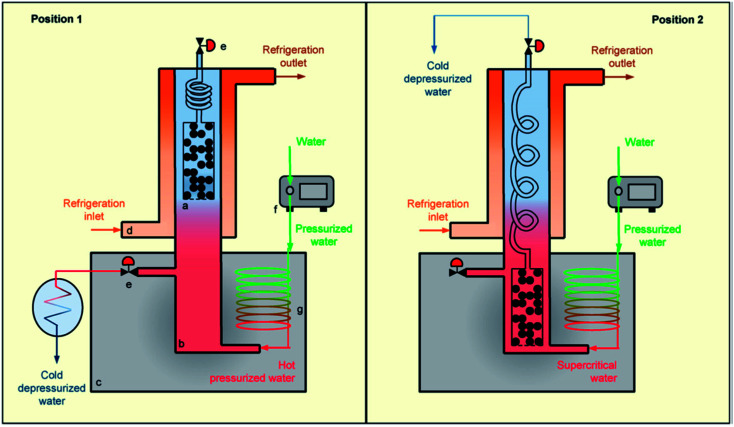
Scheme of a supercritical regeneration setup, including sample holder (a), reactor (b) and (c), oven refrigeration jacket (d), system of pressure regulating valves (e), dosing pump (g), preheater (f). This figure has been adapted/reproduced from ref. 163 with permission from Elsevier.

Supercritical fluid extraction (SFE) is the process of separating one component (the extractant) from another (the matrix) using supercritical fluids as the extracting solvent. A supercritical fluid is a substance that has been heated above and compressed beyond its critical temperature and critical pressure.^157^ The use of a supercritical fluid as a regeneration solvent of exhausted adsorbents is widely applied and is considered an alternative to solvent extraction or incineration.^158,159^ In a soil matrix, the supercritical fluid or solvent acts as a classical solvent and desorbs the pollutant. The pollutant is condensed by reducing the pressure and it can then be collected in a reduced volume. CO_2_ is the most widely used supercritical solvent because of its non-flammable, nontoxic, and inexpensive nature.^160,161^ Additionally, it has a higher rate of mass transfer and low surface tension. The extraction power of a pollutant depends on the density, low regeneration temperature, and pressure.^161^ Despite its many advantages, CO_2_ has been found have limitations due to its lower regeneration efficiency for adsorbents loaded with phenol.^162^ To overcome this problem, Salvador and coworkers used supercritical water, which could completely desorb phenol and achieved almost 100% efficiency.^163^ A schematic setup of a supercritical extraction technique using supercritical water as the solvent is shown in Fig. 6.

The regeneration of adsorbents using supercritical extraction showed that factors such as the density and viscosity of a supercritical fluid affects the extraction efficiency. A significant extraction efficiency (84%) of ethyl acetate using organoclays, and the adsorption capacity of modified clays was found to be same as that of virgin clays after regeneration.^164^ Instead of using only a supercritical fluid, a supercritical fluid with a co-solvent has also been employed to increase the polarity of the solvent to enhance the extraction efficiency toward pollutants. The extraction of phenol and 4-nitrophenol from organically modified smectite has been effectively achieved with and without a co-solvent (ethanol).^165^ The percentage recovery of phenol in the absence of the co-solvent was 73.6%, whereas after mixing in 2.5% ethanol at 70 °C and 413.6 bar, the recovery of phenol increased to 90.8%. Salgin *et al.*, also used ethanol (as co-solvent) to remove salicylic acid from organically modified bentonite.^166^ The desorption capacity of the salicyclic acid was 76 wt% without co-solvent and up to 98% (wt) with 10% (vol) ethanol. Supercritical extraction is a fast process, as fast as 4.17 min^167^ or as slow as 350 min.^163^ Supercritical water has the advantage of a very short process time (min), which significantly lowers the cost of regeneration, but it requires high pressure, which increases the cost of extraction and limits its uses to large-scale applications (as a regeneration technique) therefore, it can only be applied on a small scale.

### 2.3. Thermal degradation

**Fig. 7:**
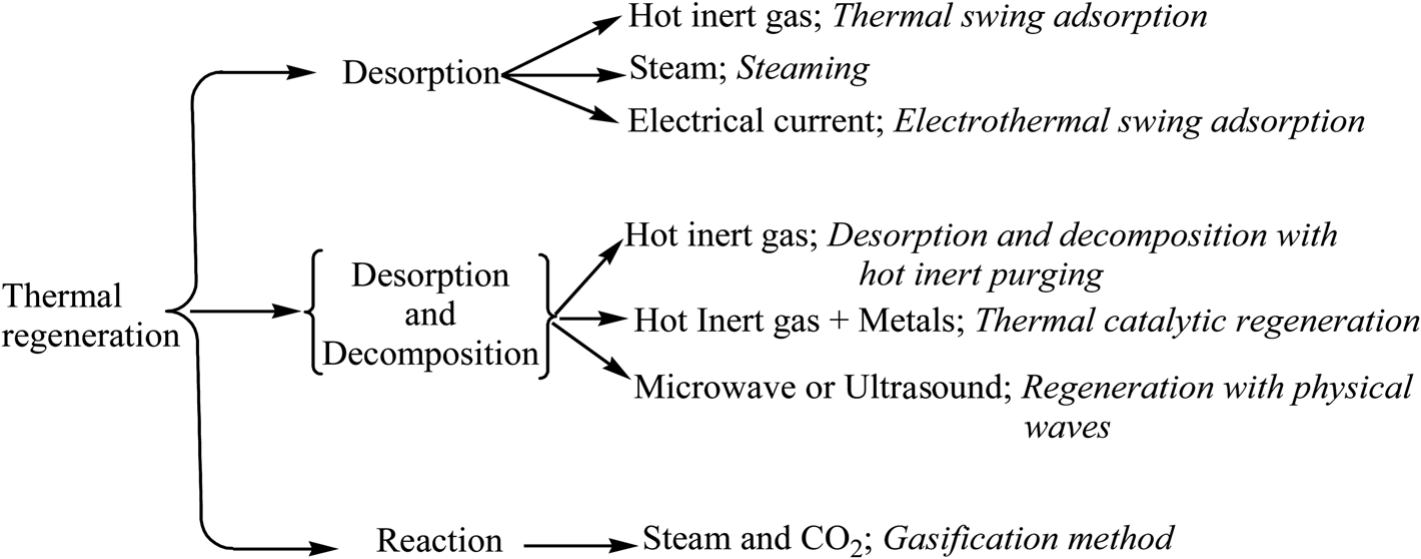
Classification of thermal regeneration methods.

Thermal regeneration involves heating an adsorbent up to a particular temperature to break the bonds between an adsorbate and adsorbent. This technique is currently used for the regeneration of activated carbon in many industries and plants. On a laboratory scale, the thermal regeneration has also been applied to regenerate exhausted clay adsorbents. The regeneration capacity of a spent clay varies with temperature and time. Lin and Cheng observed that on increasing temperature (to over 250 °C), the removal efficiency of phenol and chlorophenol decreased (Table 3).^168^ An outline for the classification of thermal regeneration methods is shown in Fig. 7.

**Table 3:** Dye removal efficiency of clay adsorbents using thermal treatment in the presence of N_2_ gas

Adsorbent	Adsorbate	Temperature (^o^C)	Removal efficiency (%)	Reference
Organobentonite	Chlorophenol	100–350	60.0	168
Clay	Oil	260–760	90.0	169
Montmorillonite	BTEX	150	51.28–60.70	170
Modified zeolite		100	77.0–92.0	171
Modified pillared clay	Phenol	500	—	172
Zeolite	Methylene blue	450	90.0	173

A mixed approach utilizing the chemical and thermal regeneration of clays, was used as fresh clay in lubricating oil refining.^169^ First, the spent clay was treated with acid to regain 55–60% its adsorption capacity. This solvent extracted clay was then heated (260–760 °C) and showed a 90% removal efficiency (Table 3). Nourmoradi *et al.* observed that the desorption efficiency of benzene, toluene, ethyl-benzene, and xylene (BTEX) was increased at higher temperatures (150 °C) in 20 min as compared to 5–10 min.^170^ In another study, Vidal *et al.* revealed a 60% removal efficiency of BTEX using a hexadecyltrimethylammonium (HDTMA) surfactant-modified synthetic zeolite at 100 °C.^171^

Thermal and Fenton oxidation methods have been used for the regeneration of exhausted/spent zeolites. Sun and coworkers regenerated zeolite by heating it at high temperatures (450 °C, 550 °C, and 650 °C); however, the optimal adsorption capacity (90–105%) was achieved at 450 °C.^173^ Thermal treatment at higher temperature may cause the loss of the structure (framework) of zeolites, resulting in a decrease in their adsorption capacity. Fenton oxidation also decomposes the adsorbent surface and pores. Ferric ions present in Fenton reagent are adsorbed or exchanged on the solid surface or pores, thus significantly reducing the ion-exchange capacity for dye re-adsorption. However, a regenerated sample obtained from air calcination showed a slightly higher adsorption than in Fenton oxidation.^174^ The thermal regeneration of adsorbents has been found to be an expensive technique due to the generation of a steam generator/inert supply to operate at high temperature, which may result in a weight loss of the adsorbent (5–15%) after every regeneration cycle. Therefore, other alternative regeneration techniques (*e.g.*, photocatalytic and biological regeneration) have been employed to regenerate and reuse spent adsorbents without any weight loss of adsorbent.

### 2.4. Photocatalytic activity

**Fig. 8:**
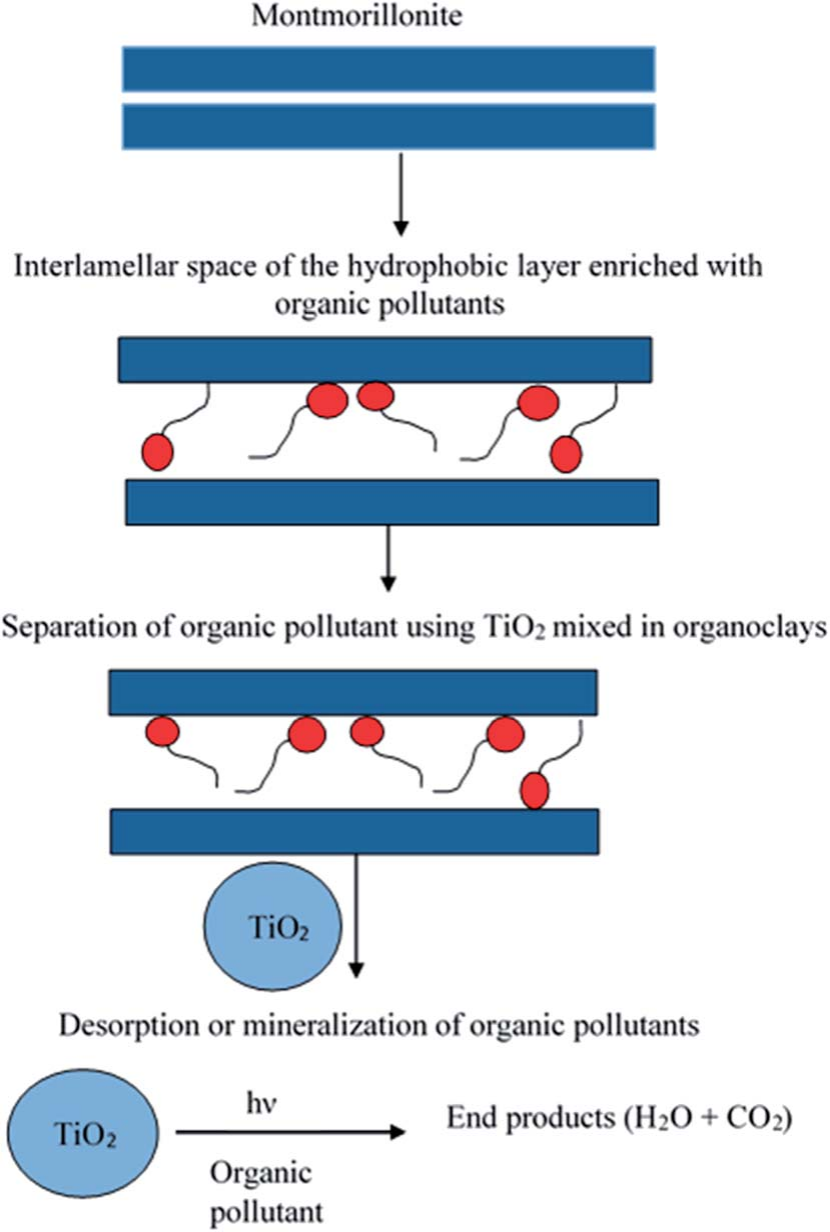
Scheme for the photocatalytic regeneration of dye pollutants using TiO_2_. This figure has been adapted/reproduced from ref. 179 with permission from Taylor & Francis.

Photocatalytic oxidation involves the oxidation of photocatalytic and photosensitizers by generating reactive free radicals to degrade various organic pollutants.^175^ This method has the potential to degrade organic pollutants down to a low concentration at a very fast rate. Photocatalyst regeneration can be performed in two ways, either by the addition of a photocatalyst semiconductor in a suspension of spent clay adsorbents^176^ or by inserting photocatalytic or photosensitizers into the intercalated layer of the clay adsorbent using UV radiation (Fig. 8). Photosensitizers displace the organic pollutants in layers and further degrade them.^177^

Metal oxides (TiO_2_ and ZnO) have been widely used as photocatalysts for the degradation of organic contaminants. They are nontoxic, inexpensive, and have been found to be an effective semiconductor.^178^ TiO_2_ has been used for the degradation of 2-chlorophenol from spent organoclays.^176,179^ At lower intensity radiation (*λ*_max_ = 254 nm), almost complete degradation (>99%) of 2-chlorophenol was observed; however, the structure of the clay was deformed. Upon increasing the radiation, a complete recovery of the adsorbents was achieved in 7 h without any structural distortion. TiO_2_ intercalated into the interlamellar space of clay was also studied.^179^ An *et al.* used photocatalytic TiO_2_ for the degradation of decabromodiphenyl ether (BDE 209) from hydrophobic montmorillonite. Almost complete removal of BDE 209 was observed after 180 min of exposure to UV irradiation at <300 nm.^180^

Photosensitizers have also been used for the degradation of organic pollutants to regenerate spent clays. One of the most widely used photosensitizers is metal phthalocyanine.^171,181,182^ Metal phthalocyanine showed higher activity under visible light irradiation (50%)^183^ as compared to the popular sensitizer porphyrin, which could reduce the overall cost of regeneration. By incorporating into the layers of a surfactant-modified clays, it can enhance the removal efficiency of phenols and organic sulfides.^177,182^ Among the metal oxides, TiO_2_ is the most popular semiconductor used in removing pollutants from wastewater. It has a high band gap energy (3.2 eV) without visible light sensitivity.^184^ Zinc oxide (ZnO) has also been chosen as a low-cost photocatalyst as it has high photocatalytic activity and covers a comparable band gap energy to TiO_2_ ^41,185^ compared to the photocatalytic activity of TiO_2_ and ZnO for the degradation of organic sulfide under UV light in a solvent medium.^186^ For the photocatalytic degradation of 2-mercaptobenzoic acid (MBA) and methyl phenyl sulfide (MPS), the order of activity was TiO_2_ (rutile) > ZnO > TiO_2_ (anatase). Photo-assisted regeneration is widely used on the laboratory scale, but no concrete evidence has been found yet showing the nontoxic results of the by-products to humans. The removal of dyes using a photocatalyst depends on the operating conditions as most dyes are resistant to photodegradation.^187^

### 2.5. Biological degradation

Microbial regeneration of an adsorbent involves renewing the adsorbent using biodegradation of the retained organics by microbial activities.^188^ It is carried out by mixing microorganisms, such as bacteria, with the saturated adsorbent. Biological degradation can be achieved either by mixing bacteria with saturated activated carbon in offline systems^189,200^ or it can be achieved in the course of biological treatments.^191,192^ In offline bioregeneration, microbe nutrients and dissolved oxygen are mixed with pollutant-loaded adsorbents in a batch system, followed by the desorption and degradation of the adsorbates on the adsorbents.^188^

The are two mechanism for the bioregeneration. The first is desorption due to the concentration gradient in which the released compound is degraded by microbial activity, which reduces the concentration of pollutants in the liquid phase. As a result, there is a concentration gradient between the adsorbent surface and bulk fluid. Differences in the Gibbs free energy of molecules in solution (−Δ*G*^0^_ads_) and molecules inside the porous structure (−Δ*G*^0^_ads_) also depends on the driving force of bioregeneration.^193^ The second mechanism is due to the discharging of exoenzymes, which diffuse in to the pores of the adsorbents and react with the adsorbates (followed by hydrolytic decay of the substrate or the desorption-resulting enzyme metabolite). Effective bioregeneration depends on a number of factors, such as the type of microbe present, the optimal microbial growth, including nutrients, temperature, dissolved oxygen, and the microbe/adsorbate concentration ratio.^194,195^

Bioregeneration for clays or modified clays was reported by Yang *et al.*, who found that the biological regeneration of hexadecyltrimethylammonium (HDTMA)-modified montmorillonite was more effective than chemical regeneration.^147^*Pityrosporum sp.* yeast was used for the regeneration of HDTMA-modified montmorillonite. It was observed that phenol was completely degraded due to the long incubation time and also that the sorption capacity could be completely recovered after the repeated biological regenerations.^196^ The major drawback associated with microbial regeneration is its low regeneration rate, which means it is not an attractive option for large-scale treatments. Furthermore, not all adsorbents are suitable for microbial regeneration. Some reagents, such as cationic surfactants, which have been used to modify adsorbents to improve the cation-exchange capacity of the adsorbent, are toxic to microbes.^197^ Some important regeneration techniques as well as the affecting factors, advantages, and disadvantages are listed in Table 4.

**Table 4:** Overview of various regeneration techniques

Techniques	Affecting parameters (factors)	Advantages	Disadvantages	Reference
Chemical treatment	• Concentration of solvents	✓ Cost effective	✓ It can modify or destroy the surface properties of adsorbents	198
	• Solubility of adsorbates			
	• Charge of adsorbents	✓ Fast regeneration	✓ Production of oxidized sludge/wastage	
	• Solution pH			
Supercritical fluid extraction	• Different types of supercritical fluids	✓ Very short process time	✓ High pressure	167
	• Temperature			
	• Pressure		✓ Applicable mostly on a small scale	
	• Pollutant solubility			
Thermal degradation	• Heating time and temperature of adsorbent	✓ It is useful for the adsorbents which are loaded with heterogeneous adsorbate	✓ Requires high temperature	198
	• Type of adsorbate and adsorbent		✓ Weight loss after every regeneration cycle	199
			✓ Release of harmful gases during heating causing air pollution	200
Photo-assisted activity	• Type of photocatalyst and photosensitizer	✓ Fast removal of pollutants down to very low concentration	✓ Generation of by-products	187
		✓ Ecofriendly		177
Biological treatment	• Nature of adsorbent	✓ Converts the toxic organic pollutant into small ionic toxicants, which helps the adsorbent be regenerated completely	✓ Only applicable to biodegradable pollutants and not suitable for modified adsorbents	201
	• Concentration of adsorbate		✓ Regeneration is very slow	191
	• Types of microorganisms		✓ Fouling can occur in the pores of adsorbents by microbial activity	147
	• Optimal microbial growth condition			

## 3. Critical comparison of regeneration techniques

Adsorption is the most adaptable and widely used method for water treatment because of its cost-effective and feasible nature compared to other methods. Recently, a thin layer of clay polymer adsorbent coating was found to be quite effective in the treatment of environmental pollutants to overcome the problems associated with adsorbents used in the form of pellets, beads, powder, or other particles. The thin-coated layer techniques increased the surface area and hence the adsorbents possessed a higher adsorption capacity. To use exhausted clay adsorbents for further treatment, regeneration plays an important role to help them regain their adsorption capacity. Various techniques, including chemical, thermal, photocatalytic, and biological approaches, have been applied for stimulating spent adsorbents. Chemical regeneration using an oxidation method (*e.g.*, Fenton oxidation) is supposed to be an effective approach for the degradation of organic pollutants. However, the toxicity of unknown by-products is an issue with chemical and photocatalytic regeneration techniques. Chemical desorption (regeneration) methods can be controlled by treating the adsorbents in an inert atmosphere.

Supercritical regeneration extraction needs high pressure, which increases the cost of extraction, thus appropriate techniques are being tested on pilot or large-scale applications. A thermal technique for the regeneration of modified clays was found to be effective; however, it could cause a loss of adsorbents, which eventually leads to a reduction in regeneration efficiency. Furthermore, it is an expensive technique due to the higher temperature and high cost of equipment for thermal treatment. Biological (microbial) regeneration has the potential to stimulate spent adsorbents; however, the low rate of regeneration has restricted it to so far to industrial-scale dye treatment. Moreover, not all adsorbents are suitable for microbial regeneration owing to the use of certain reagents (*e.g.*, cationic surfactants to improve the exchange capacity) of modified adsorbents, which have been found to be toxic to microbes.

Among all the regeneration techniques, no stimulating technique has been individually found to retain or improve the adsorption efficiency of all adsorbents, especially for clay-modified adsorbents. Some particular techniques have been established for specific adsorbents though. Combinations of one or more regeneration techniques may also be effective and an alternative to stimulate spent adsorbents. Regeneration techniques depend on the nature and type of adsorbate and adsorbent (*e.g.*, toxicity, combustible, corrosive, and radioactive, physical adsorption or chemisorption). Thus, the adopted regeneration techniques should be efficient, nontoxic, ecofriendly, cost effective, easy to operate, and give the ability to reuse the stimulated spent adsorbent in water treatment.

## 4. Future perspectives and conclusions

A number of studies related to the adsorption behavior of different adsorbents for the removal of dyes from wastewater have been published and discussed herein. Clay-supported adsorbents were found to be more effective than activated carbon, and zeolites organic/inorganics, and hybrid materials for dye treatment. Modified clay with a thin-coated layer of adsorbent proved to be more effective than pure clay. Much research has focused on the modification of clay owing to being able to achieve a better porosity and higher adsorption capacity. Modified clay with a high surface area has been shown to have better selectivity for organic pollutants. Regeneration techniques play a key role to enable reuse of spent adsorbents for the treatment of wastewater. The extent of accessible records for the regeneration of the adsorbent is reasonably inadequate in comparison to the modification or fabrication of clay adsorbents. Most of the spent adsorbents regeneration studies have been carried out only on a lab scale, which is far away from the real image of how adsorbents perform in practice in water treatment. Degradation of the spent adsorbent, a long process time, expensive process (need to maintain at high temperature), complex steps, and slow rate of regeneration techniques have been found as the main bottlenecks to the reuse of adsorbents in pilot plants. On top of applying the above-mentioned steps for the regeneration of organic/inorganic and hybrid adsorbents, the production of sludge is another environmental issue. However, the sludge production problem can be tackled by using clay-based regenerated adsorbents or an adsorbent coating owing to their ecofriendly nature. On the basis of the effective adsorption capacity of clay-based adsorbents, it can be expected that these low-cost adsorbents and adsorbent coatings will rapidly develop their own adsorption/desorption characteristics to support a better pollution-free green environment. The present review states highlights how clay-based low-cost adsorbents can clearly be considered as smart materials along with their recyclability for the removal and recovery of dye pollutants from waste waters. Consequently, clay-based adsorbent may open a new approach in the form of instructive agents to generate a pollution-free environment in the treatment of industrial dyes. Hence, future work should be focused on the areas of developing and utilizing clay-based adsorbents.

## Conflicts of interest

There are no conflicts to declare.

## Supplementary Material
